# Prevalence and risk factors of functional seizures among adult Sudanese patients with epilepsy, a cross-sectional clinic-based study

**DOI:** 10.1016/j.amsu.2022.104712

**Published:** 2022-09-16

**Authors:** Khabab Abbasher Hussien Mohamed Ahmed, Walaa Elnaiem, Yassin Abdelrahim Abdalla, Salih Boushra Hamza, MuazA. Ibrahim, Abdallah M. Abdallah, Mawahib Hajhamed, Ghassan Elfatih, Aziza Fakhreldeen, Tibyan Hassan, Roaa Faisal, Rufaida A. Salih, Mihad A. Mahmoud, Mwaez Ahmed, Yousif Fadlallah, Radi Tofaha Alhusseini, Nijood Albasheer, Lina Shamsaldeen, Leenah Mohammed, Amira Siddig, Hussam Mohamedalhadi Alamin Alkhalifamohamed, Esraa Hassan Salih, Abbasher Hussien, Mohammed Mahmmoud Fadelallah Eljack

**Affiliations:** a University of Khartoum, Faculty of Medicine, Khartoum, Sudan; b Omdurman Islamic University, Faculty of Medicine, Khartoum, Sudan; c Omdurman Islamic University, Faculty of Medicine, Omdurman, Sudan; d University of Bahri, Faculty of Medicine, Teaching Assistant at Department of Internal Medicine, Khartoum North, Sudan; e University of Bahri, Faculty of Medicine, Police Hospital, Khartoum, Sudan; f Ahfad University for Women, Faculty of Medicine, Khartoum, Sudan; g University of Khartoum, Faculty of Medicine, Omdurman, Sudan; h Ahfad University for Women, Faculty of Medicine, Omdurman, Sudan; i Ahfad University for Women, Faculty of Medicine, Khartoum North, Sudan; j Alzaiem Alazhari University, Faculty of Medicine, Khartoum, Sudan; k Al Neelain University, Faculty of Medicine, Khartoum, Sudan; l Community Department, University of Bakht Alruda, Ad Duwaym, Sudan

**Keywords:** Functional seizures, Psychogenic non-epileptic seizures, Pseudo epilepsy, Epilepsy, Stigma

## Abstract

**Background:**

Epilepsy can be associated with functional seizures. Our main aim is to assess functional seizures' prevalence and risk factors among adult Sudanese patients with epilepsy.

**Methods:**

This cross-sectional clinic-based study was conducted from January to February 2021 at Daoud Charity Clinic in Omdurman city, Sudan. Ninety-nine adult Sudanese patients with epilepsy were included. Data were collected using a validated interview-based semi-structured questionnaire. A senior consultant neurologist and a consultant psychiatrist diagnosed the functional seizures based on full clinical history and investigations. The diagnosis was performed according to International League against epilepsy (ILAE) classification.

**Results:**

This study included 99 patients with epilepsy, 57% were females, 79% reside in Khartoum state, and 32% reached secondary school. The main types of epilepsy were generalized tonic-clonic (68%), followed by focal seizures with impaired awareness (11%). The majority of the patients have been diagnosed with epilepsy for over three years (65%). Comorbid epilepsy and functional seizures were found in 29% of the patients, with a significantly higher prevalence in patients with social problems and depression (p = 0.005 and p < 0.01, respectively). Patients with depression had a 14 times higher risk of functional seizures than those without depression, 95% CI [3.8, 52.3].

**Conclusion:**

A remarkably high prevalence of functional seizures was found among adult patients with epilepsy. Patients suffering from social problems and/or depression and poor economic status had a higher tendency to develop functional seizures, especially after two to three years of treatment and above.

## Highlights

• Epilepsy is among the most familiar stigmatizing diseases in our community.
• high prevalence of functional seizures was found among adult patients with epilepsy.
• low socioeconomic status and depression are the higher risk factors for functional seizures.
• The present study has shed light on the fact that functional seizures should be primarily recognized by the treating doctor .

## 1 Introduction

Epilepsy is characterized by a persistent tendency to experience epileptic seizures [1]. Epilepsy diagnosis depends on careful history taken from good eyewitnesses [2]. The International League against epilepsy (ILAE) divided epilepsy into four categories: generalized epilepsy, focal epilepsy, combined generalized/focal epilepsy, and an unknown category [3]. The management plan focuses on controlling seizures, avoiding the drugs' side effects, and maintaining the quality of life [4]. Functional seizures clinically resemble epileptic seizures, but without physiological central nervous system dysfunction [5]. The best diagnostic tool available for functional seizures is Video-EEG [6]. Stigma perceived by the patients with epilepsy can be classified into enacted, felt in patients, and coaching and courtesy in their families [7]

The coexistence of epilepsy and functional seizures places a great challenge for psychiatrists and neurologists in following up with their patients, as functional seizures can be misdiagnosed as epileptic seizures. Before consulting their neurologist, almost all patients' relatives consider functional seizures as epileptic seizures. This misunderstanding can create or increase epilepsy stigma in the patients and their families. Additionally, the patients and/or their families may lose hope in medical therapy and seek help from traditional healers (ex. herbal remedy and Sheikh).

Functional seizures are not uncommon where estimates of 2–50/100,000 in the general population. However, the prevalence is higher among patients with epilepsy. The prevalence is 20–40% among inpatients in the monitoring units of epilepsy and 5–10% among outpatients [8,9]. The lack of appropriate diagnosis of functional seizures among patients with epilepsy can result in increasing anti-epileptic drug doses in a potentially harmful way, as well as making the diagnosis of refractory epilepsy [10]. In patients admitted to monitoring units of video-EEG for diagnosing drug-resistant epilepsy, 20% were found to be suffering from functional seizures [11]. According to the ILAE, functional seizures are among the top ten critical neuropsychiatric conditions [11]. Functional seizures' presence is usually attributed to financial, interpersonal, and psychiatric problems [12]. Depression, anxiety disorders, and relationship problems substantially impact the quality of life. These disorders are more prevalent among patients with functional seizures when compared to the general population and even those with epilepsy [8]. Long duration, fluctuating course, asynchronous movements, pelvic thrusting, side-to-side head or body movements, ictal eye closing, ictal crying, memory recall, and absent postictal confusion were the most reliable signs in distinguishing functional seizures from epileptic ones [5]. Stigma is another problem encountered in both types, but the perceived stigma risk experience is 42% higher in patients with functional seizures than in patients with epilepsy [13].

Globally, this area of coexistence between epilepsy and functional seizures is not that clear and has not been researched thoroughly [14]. There must be a clear literature base regarding the relation between functional seizures and epilepsy to avoid mixing the two conditions by the patients or their relatives and prevent further neurologists' misdiagnosis. By the end of this research, we expected to find an estimated prevalence of functional seizures among adult Sudanese patients with epilepsy and its relationship with other biopsychosocial factors.

## 2 Methods and materials

### 2.1 Study setting and participants

This Descriptive cross-sectional facility-based study was conducted at Daoud charity clinic from January to February 2021. There was a clear observation that patients with epilepsy who regularly attend Daoud charity clinic start developing functional seizures following their initial diagnosis of epilepsy. Therefore, to study this comorbidity, we included all Sudanese adult patients with epilepsy who attended Daoud charity clinic during the time of the study. Patients less than 18 years old and those who refused to participate were excluded from our sample. A senior consultant neurologist and a consultant psychiatrist took full history and investigations (including EEG and brain MRI). Diagnosis of functional seizures was confirmed by conducting video-EEG studies that provide a documented diagnosis, representing the most reliable diagnostic level according to LaFrance et al. [15]. Furthermore, a psychiatric evaluation was made by the consultant psychiatrist. Anxiety was diagnosed according to the Generalized Anxiety Disorder (GAD-7) screening tool, and depression was diagnosed according to the Diagnostic and Statistical Manual of Mental Disorders (DSM-5).

Daoud charity clinic was commenced as a Neurological clinic in 1995, serving 150–200 patients with epilepsy per week from all over Sudan. A senior consultant neurologist supervises the clinic. Under him, registrars work, and medical students attend the neurological history and examinations. A senior psychiatrist is also involved and is consulted in cases of functional seizures and psychiatric disorders.

This study is fully compliant with the STROCSS 2021 criteria [16].

### 2.2 Data collection

Data were collected using a well-constructed, semi-structured, highly confidential questionnaire containing close-ended questions. The questionnaire consisted of seven sections; socio-demographic, history of epilepsy, social history, stigma, history in favor of functional seizures, psychiatric history, and investigation (EEG, MRI).

The collected demographic data included age, gender, occupation, residency, educational level, and monthly income. The detailed history of epilepsy included the date of first and last attack, Seizure type, frequency of attacks, seizure-free period, past medical history, and social and drug history. The history of functional seizures included stress, the time between the attack and stress, description of the attack in favor of functional seizures, duration of the attack, and sexual and physical abuse. Moreover, the participants were asked about their social history, type and duration of epilepsy, and type and number of anti-epileptic drugs. Enacted stigma, courtesy and coaching stigma, and coping strategies were evaluated using the standardized questionnaire developed by Bashir et al. [17]. Enacted stigma assessed the experienced discrimination and social reaction to the patient's condition. Whereas courtesy stigma evaluated the relatives' feeling of shame, and coaching stigma evaluated whether the patients received negative assertiveness from their relatives [17]. Furthermore, the relevant available investigation findings (brain MRI and EEG) were taken. The diagnosis was performed according to the ILAE classification.

### 2.3 Data analysis

Data were analyzed using IBM statistical package of social science software version 26 (SPSS 26) and Microsoft office excel. Continuous data were summarized as mean and standard deviation, whereas categorical data were summarized as frequencies and proportions. A Chi-square test was conducted to compare the frequency of categorical variables between patients who experienced functional seizures and those who did not. Binary logistic regression was used to determine the relationship between the occurrence of functional seizures and other factors. Analysis was conducted with a 95% confidence interval, and a p-value < 0.05 was considered statistically significant.

Ethical consideration and Patients' consent:

Ethical approval was obtained from the Sudan State Ministry of Health. Both privacy and protection of the participant's files and information were of the highest priority. Written and verbal informed consent were taken from the participants.

## 3 Results

1. Demographic characteristics

The sample consisted of 99 adult patients with various types of epilepsy. The mean age of the patients was 31 ± 13.6 years, 57% were females, and the majority of patients reside inside Khartoum and reached secondary school (79% and 32%, respectively). The majority of participants were single (63%), and had a monthly income of <5000 SDG (= 12.5 US dollars, and is below the poverty line) (Table 1). Participants were of different occupations. About 29 patients (29.3%) had a history of functional seizures.

2. History of epilepsy

**Table 1:** Demographic characteristics and its association with functional seizures.

Demographic characteristics		History of functional seizures		Total n (%)	P-value
		No, n (%)	Yes, n (%)		
Gender	Female	38 (67.9%)	18 (32.1%)	56 (56.6%)	0.4
	male	32 (74.4%)	11 (25.6%)	43 (43.4%)	
Residency	Khartoum state	51 (65.4%)	27 (34.6%)	78 (78.8%)	0.02*
	Outside Khartoum state	19 (90.5%)	2 (9.5%)	21 (21.2%)	
Marital status	Divorced	2 (66.7%)	1 (33.3%)	3 (3.0%)	0.9
	married	24 (70.6%)	10 (29.4%)	34 (34.3%)	
	Single	44 (71%)	18 (29%)	62 (62.6%)	
Monthly income	<5000	27 (58.7%)	19 (41.3%)	46 (46.5%)	0.03*
	5000 and less than 10000	23 (76.7%)	7 (23.3%)	30 (30.3%)	
	≥10000	20 (87%)	3 (13%)	23 (23.2%)	

The main types of epilepsy were generalized tonic-clonic (68%), followed by focal with impairment (11%). The majority of patients (65%) have been diagnosed with epilepsy for over three years. About 20% of participants completed three years treatment-free period without attacks, among them 56.7% had a recurrence thereafter (Table 2). Most of the participants (60%) did not have a known risk factor for epilepsy, and 72% had no family history of epilepsy. About 67% of the participants were on sodium valproate either alone (50%) or in combination with other drugs, and 39% were on carbamazepine. A minority of the participants were on Levetiracetam or lamotrigine. Around 51% of the participants were on treatment for a minimum of three.

3. Psychiatric and Social problems and stigma of epilepsy

**Table 2:** Relationship between characteristics of epilepsy and functional seizures.

Variable		History of functional seizures				P-value
		No, n (%)		Yes, n (%)		
Types of epilepsy	One type	69 (98.6%)	27 (93.1%)		0.20	
	More than one type	1 (1.4%)	2 (6.9%)			
Duration of epilepsy (years)	<1	7 (10%)	1 (3.4%)		0.36	
	1 and less than 2	12 (17.1%)	2 (6.9%)			
	2 and less than 3	43 (61.4%)	21 (72.9%)			
	≥3	8 (11.4%)	5 (17.2%)			
Treatment Free attack for three years	No	53 (75.7%)	26 (89.7%)		0.09	
	Yes	17 (24.3%)	3 (10.3%)			
Recurrence of epilepsy after Treatment Free attack for three years	No	7 (100%)	10 (76.9%)		0.09	
	Yes	0 (0%)	3 (23.1%)			
Known risk factors of etiology for epilepsy	No	42 (60%)	17 (58.6%)		0.89	
	Yes	28 (40%)	12 (41.4%)			
Family history for epilepsy	Yes	50 (71.4%)	21 (72.4%)		0.92	
Drug history	No	20 (28.6%)	8 (29.3%)		0.61	
	One drug	54 (77.1%)	21 (72.4%)			
	More than one drug	16 (22.9%)	8 (27.6%)			
Duration of treatment (years)	<1	16 (22.9%)	0 (0%)		0.017*	
	1 and less than 2	11 (15.7%)	4 (13.8%)			
	2 and less than 3	33 (47.1%)	17 (58.6%)			
	≥3	6 (8.6%)	6 (20.7%)			
	Did not start treatment yet	4 (5.7%)	2 (6.9%)			
MRI finding	Normal	37 (17.8%)	8 (82.2%)		0.12	
	Abnormal	13 (65%)	7 (35%)			
EEG finding	Normal	23 (79.3%)	6 (20.7%)		0.26	
	Abnormal	17 (68%)	8 (32%)			

Among the included patients, 28% had a history of depression, and 19% had a history of anxiety. About 37% of the patients had social stressors. Among these stressors, problems with school, university, or work were the most frequent (55%), followed by problems with friends and relatives (43%). Regarding stigma, 61% of the patients did not have enacted stigma. Regarding courtesy and coaching stigma, 74% had no stigma, 16% had a moderate stigma, and 10% had a severe stigma. Furthermore, 62% had poor coping abilities, as they were using escape-avoidance and distancing coping strategies (Table 3).

4. Functional seizures association with demographic characteristics

**Table 3:** Functional seizures association with social and psychiatric problems.

Social and psychiatric problem		History of functional seizures		Total n (%)	P value	
		No, n (%)	Yes, n (%)			
Presence of Social problems	No	50 (50.5%)	12 (12.1%)	62 (62.6%)		0.005*
	Yes	20 (54.1%)	17 (45.9%)	37 (37.4%)		
History of depression	No	60 (84.5%)	11 (15.5%)	71 (71.7%)		<0.01*
	Yes	10 (35.7%)	18 (64.3%)	28 (28.3%)		
History of anxiety	No	60 (75%)	20 (25%)	80 (80.8%)		0.054
	Yes	10 (52.6%)	9 (47.4%)	19 (19.2%)		
Enacted stigma	No	44 (72.1%)	17 (27.9%)	61 (61%)		0.6
	Yes	26 (68.4%)	12 (31.6%)	38 (38%)		
Courtesy and coaching stigma	No	55 (75.3%)	18 (24.7%)	73 (73%)		0.2
	Moderate	10 (62.5%)	6 (37.5%)	16 (16%)		
	Severe	5 (50%)	5 (50%)	10 (10%)		
Coping grade	Good	30 (81.1%)	7 (18.9%)	37 (37%)		0.08
	Poor	40 (64.5%)	22 (35.5%)	62 (62%)		

There was no significant relation between functional seizures and gender or marital status. However, patients residing in Khartoum state had significantly higher frequencies of functional seizures (p = 0.02). As the monthly income of the participants decreased, the frequency of functional seizures significantly increased, which means the financial status was inversely proportional to the frequency of functional seizures (p = 0.03). (Table 1).

5. Relationship between characteristics of epilepsy and functional seizures

Most of the patients who have been treated with anti-epileptic drugs for two to three years did not have functional seizures (Table 2).

6. Functional seizures associated with social and psychiatric problems and stigma

The prevalence of functional seizures was significantly higher in patients with social problems and depression (p = 0.005, <0.01 respectively). Patients with depression had 14 times the risk of developing functional seizures compared to those without depression. No significant relation was detected between any type of stigma and functional seizures (Table 3).

7. History in favor of functional seizures

Most of the participants did not have a history of sexual abuse, physical abuse, or school/University/work refusal and\or absence (94%, 84%, and 63%, respectively); while 69% had a history of traumatic brain injury (Table 4). No significant relation was detected between the history of school/University/work refusal and/or absence and functional seizures (p = 0.7). In addition, no relation between the history of traumatic brain injury and functional seizures was found (p = 0.7) (Table 5).

**Table 4:** Functional seizures association with history in favor of functional seizures.

History in favor of functional seizures			History of functional seizures				Total n (%)	P value
			No, n (%)		Yes, n (%)			
History of school/university/work refusal and\or absence	No	43 (69.4%)		19 (30.6%)		62 (62.6%)		0.7
	Yes	27 (73%)		10 (27%)		37 (37.4%)		
History of traumatic brain injury	No	48 (71.6%)		19 (28.4%)		67 (67.7%)		0.7
	Yes	22 (68.8%)		10 (31.2%)		32 (32.3%)

**Table 5:** Predictors of functional seizures.

Predictors	EXP (B)	P value	95% CI for EXP(B)		
Physical abuse	0.514	0.38		0.11	2.33
Sexual abuse	0.896	0.92		0.09	8.9
depression	14.102	0.00*		3.8	52.3
Anxiety	1.548	0.54		0.37	6.4
psychosis	0.863	0.87		0.14	5.17

## 4 Discussion

This study was directed to determine the functional seizures prevalence and its associated biopsychosocial aspects among known patients with epilepsy. Ninety-nine patients were recruited, and 29% of them showed a positive history of functional seizures, which is much higher than the 5–10% prevalence reported in the literature [9]. According to a systematic review by Gislaine Baroni et al., in 2016, patients diagnosed with functional seizures had a history of epilepsy that preceded the functional seizures. Although psychiatric symptoms are usually associated with temporal lobe epilepsy, they can be found in other epileptic conditions. Abnormalities in brain structure may increase the risk not only for epilepsy but also for other cognitive and psychiatric disorders [14,18]. Although 57% of the patients in our study were females, the association between functional seizures and gender was not statistically significant, unlike the literature findings. In a study conducted in 2016 by Asadi-Pooya, functional seizures were more prevalent among females [9]. Similarly, marital status was not related to functional seizures in our study. Most of the enrolled patients reside in Khartoum state. Among those patients, 34.6% showed a positive history, which generates a statistically significant relationship between living in Khartoum state and having functional seizures (p = 0.02). This attribute could be because of the state urbanization.

Interestingly patients who live in low socioeconomic conditions with a salary of <5000 SDG per month (= 12.5 US dollars, below the poverty line) had a significantly higher frequency of functional seizures (p = 0.03). Considering the history of epilepsy, generalized tonic-clonic was the most frequent type with a prevalence of 68%, followed by focal with impairment in 11% of the patients. A predominant number of these patients have not completed three years free of fits. Regarding the psychiatric problems, there was no history of depression or anxiety among most of the enrolled patients. Similarly, social problems were not implicated in most of the patients. Therefore, functional seizures could be associated with psychiatric and social traumas. Concerning courtesy and coaching stigma, 16% of the patients suffered from moderate stigma while 10% had a severe stigma, with 62 of the 99 patients having poor coping scores. These findings coincide with another interesting Sudanese study conducted in 2017 [17]. Our current study found no significant association between functional seizures and type and duration of epilepsy, family history, or drug history. However, there was a significant association between experiencing functional seizures and the treatment duration (p = 0.007). About 58.6% of those who experienced functional seizures were in two to three years of treatment. Those in the first year of treatment were unlikely to have functional seizures. Another important finding was the insignificant association between MRI and EEG finding and experiencing functional seizures. A better understanding of functional seizure symptomatology is needed to diagnose functional seizures earlier and more accurately [19].

The prevalence of functional seizures was high among patients with depression and social problems (p < 0.05). We suggest that this prevalence may be explained by the poor coping ability of those patients (62%), as they were using escape-avoidance and distancing strategies. A previous study reported that patients with functional seizures tend to utilize significantly more escape–avoidance and distancing coping strategies than the control healthy group. The study also indicated that this avoidance of coping by patients with functional seizures has significantly affected their quality of life. Thus, our findings support the notion that patients with functional seizures do not prefer to approach stressful situations, instead they prefer to avoid them. This avoidance behavior may increase the risk of developing functional seizures [20].

Epilepsy is among the most familiar stigmatizing diseases in our community. This stigma affects the lifestyle and mental health of patients specifically. Our study found that 28% of patients with epilepsy had a history of depression, 18% had a history of anxiety, and 37% complained of social problems, especially in school and work (55%). Additionally, 26% had a stigma of epilepsy, and 10% had a severe stigma. The mentioned prevalence of stigma among patients with epilepsy in the literature can arguably contribute to the high prevalence of functional seizures. In a study conducted by a consultant neurologist et al. (2012) among Sudanese patients with epilepsy and their relatives, 16.3% of the patients had positive felt-stigma scores. Almost half of those patients experienced courtesy and coaching. Our study observed a high prevalence of stigma among patients with epilepsy and those with comorbid epilepsy and functional seizure. Interestingly, this finding highlights the high level of stigma in our community for patients with seizures of any type. A poor coping score was found in one-fifth of patients with epilepsy. A determinant factor that is important for patients to cope with epilepsy and courtesy stigma was age below forty. Having more than three seizures per month reduces the coping score of patients with epilepsy [7]. An exploratory study found that the risk of developing perceived stigma in patients with functional seizures was 43%, which is greater than that in epilepsy. Seizure frequency, depression, anxiety, and many sequels of the condition were associated significantly with perceived stigma in epilepsy but not in functional seizures [13]. On the other hand, the findings of our study showed no significant direct relationship between stigma and functional seizures. This might be attributed to the religious and cultural background of the Sudanese community, as they perceive such diseases as a test of faith and strength from God. Yet, stigma can greatly contribute to the previously mentioned depression and anxiety, as these, in turn, form a risk factor for developing functional seizures among patients with epilepsy.

## 5 Conclusion

Returning to the question targeted at the beginning of this study, it is now possible to state that there is a high prevalence of functional seizures among adult patients with epilepsy (29%). Functional seizures can be highly associated with depression, anxiety, low socioeconomic status, and the last two years of treatment. When treating patients with epilepsy, stigma must be considered a remarkable predisposing factor for developing functional seizures. The present study has shed light on the fact that functional seizures should be primarily recognized by the treating doctor and considered one of the top differentials as it has a high rate of occurrence and should not be overlooked.

## Recommendations

● From what was mentioned above, we strongly urge decision-makers to provide psychological health services to all patients of epilepsy, especially those who are on chronic use of medications. These services should include follow-up by psychologists routinely to pick up early risk factors of functional seizures and treat them earlier.
● We also recommend including and enrolling adult patients with epilepsy in social programs (e.g., health insurance, rehabilitation programs, developing epilepsy associations, etc.).
● Since this is the first research focus/ing on the relationship between epilepsy and functional seizures, we recommend conducting more studies in the same field, especially in developing countries. These studies are especially needed due to the absence of awareness about epilepsy, and psychological and psychiatric support for patients with epilepsy, their relatives, and the surrounding community.

## Ethical approval

Ethical approval was obtained from the Sudan State Ministry of Health. Both privacy and protection of the participants' files and information were of the highest priority.

## Sources of funding

The study was self funded.

## Author contribution

All authors contributed significantly to this work.

## Registration of research studies

1. Name of the registry:
2. Unique Identifying number or registration ID:
3. Hyperlink to your specific registration (must be publicly accessible and will be checked)

## Guarantor

Mohammed Mahmmoud Fadelallah Eljack.

University of Bakht Alruda, Faculty of Medicine, Ad Duwaym, Sudan.

Wad medani, Gezira State, Postal code: 11112, Sudan, Mobile: 00249964656914

m.mahmmoud96@gmail.com.

ORCID: 0000-0002-2370-9368.

## Consent

Both written and verbal consents were taken from each patient.

## Provenance and peer review

Not commissioned, externally peer-reviewed.

## Declaration of competing interest

All authors declare that there are no conflicts of interest.
